# Drug-drug interactions and adverse drug reactions in polypharmacy among older adults: an integrative review**^1^**

**DOI:** 10.1590/1518-8345.1316.2800

**Published:** 2016-09-01

**Authors:** Maria Cristina Soares Rodrigues, Cesar de Oliveira

**Affiliations:** 2 PhD, Associate Professor, Departamento de Enfermagem, Faculdade de Ciências da Saúde, Universidade de Brasília, Brasília, DF, Brazil. Scholarship holder from Coordenação de Aperfeiçoamento de Pessoal de Nível Superior (CAPES), Brazil.; 3 Researcher, Departament Epidemiology and Public Health, University College London, London, United Kingdom.

**Keywords:** Aged, Polypharmacy, Evidence-Based Practice, Review

## Abstract

**Objective:**

to identify and summarize studies examining both drug-drug interactions (DDI) and adverse drug reactions (ADR) in older adults polymedicated.

**Methods:**

an integrative review of studies published from January 2008 to December 2013, according to inclusion and exclusion criteria, in MEDLINE and EMBASE electronic databases were performed.

**Results:**

forty-seven full-text studies including 14,624,492 older adults (≥ 60 years) were analyzed: 24 (51.1%) concerning ADR, 14 (29.8%) DDI, and 9 studies (19.1%) investigating both DDI and ADR. We found a variety of methodological designs. The reviewed studies reinforced that polypharmacy is a multifactorial process, and predictors and inappropriate prescribing are associated with negative health outcomes, as increasing the frequency and types of ADRs and DDIs involving different drug classes, moreover, some studies show the most successful interventions to optimize prescribing.

**Conclusions:**

DDI and ADR among older adults continue to be a significant issue in the worldwide. The findings from the studies included in this integrative review, added to the previous reviews, can contribute to the improvement of advanced practices in geriatric nursing, to promote the safety of older patients in polypharmacy. However, more research is needed to elucidate gaps.

## Introduction

The world is on the brink of a demographic milestone. In about five years' time, the number of people aged 65 or older will outnumber children under age 5. Driven by falling fertility rates and remarkable increases in life expectancy, population ageing will continue, even accelerate. The number of people aged 65 or older is projected to grow from an estimated 524 million in 2010 to nearly 1.5 billion in 2050, with most of the increase in developing countries^1^.

Ageing, one of the most complex biological phenomena, is a multifaceted process in which several physiological changes occur at both the tissue and the whole-organism level, occurring in cascade, especially post-reproduction^2^. The changes characterized by ageing include: changes in biochemical composition of tissues; progressive decrease in physiological capacity; reduced ability to adapt to stimuli; increased susceptibility and vulnerability to disease and increased risk of death^3^. 

Age related chronic diseases such as dyslipidemia, hypertension, diabetes, and depression usually require the use of multiple drugs, a state known as polypharmacy. This refers to the use of multiple medications and/or more medications than clinically indicated. It is estimated that more than 40% of adults aged 65 or older use 5 or more medications, and 12% use 10 or more different medications(4). However, the magnitude of the problem among older adults is still scarcely known in most countries.

It is well known in the literature that polypharmacy increases the use of inappropriate drugs, leading to the underuse of essential medicines for the appropriate control of conditions prevalent in the older adults. In addition, it sets up a barrier to treatment adherence in that it creates complex therapeutic regimens, and enables the occurrence of medication errors, drug-drug interactions, adverse reactions, and poor quality of life. It increases morbidity, mortality, and complexity of care. It also imposes a huge financial burden on both the older adults and health system^5^. 

Furthermore, attention should be paid to the fact that the body of the older adults presents changes in their physiological functions that may lead to a differentiated pharmacokinetics and greater sensitivity to both therapeutic and adverse drug effects^5^. Pharmacokinetics, pharmacodynamics, and clinical outcomes are affected by a number of patient-specific factors, including age, sex, ethnicity, genetics, disease processes, polypharmacy, drug dose and frequency, social factors, and many other factors^6^. 

The scenario above highlights that population ageing is a global phenomenon and the practice of polypharmacy is dangerous for patients, in particular for older adults, because favors the emergence of drug-drug interactions (DDI), adverse drug reactions (ADR), side effects, longer hospital stays, iatrogenic disease and may also lead to complications that induce the patient's death. Thus, the purpose of the present study was to conduct a broader integrative review aimed at identifying and summarizing studies examining both DDIs and ADRs in older adults polymedicated.

## Methods

The stages of this integrative review include: problem identification, formulating the appropriate question to be investigated; literature search with selection of articles according to predetermined criteria; data evaluation extracting data from each study summary of results; data analysis and presentation of results^7^. The following describes the steps of the integrative review for this study.

To elaborate the guiding question was applied to the PICO strategy defining population "older adults", intervention "use of multiple medications/polypharmacy" and outcome "occurrence of drug interactions and adverse drug reactions". Thus, the central question of this integrative review was: What is the scientific evidence available, demonstrating the occurrence of drug-drug interactions and adverse drug reactions in older (i.e. ≥60 years of age) polymedicated adults? 

For the selection of articles, studies published in the English, Spanish and Portuguese languages in the period between January 2008 and December 2013 were eligible. The time period was based on the existence of two previous literature reviews. One in which investigated observational studies examining the epidemiology of polypharmacy, reviewing randomized controlled studies that have been published in the past 2 decades (from 1986 until June 2007) designed to reduce polypharmacy in older adults^8^; and, another review who reviewed the body of literature addressing polypharmacy in individuals aged 60 years and older, between January 1991 and October 2003, to (a) determine primary care providers' definition of polypharmacy, (b) explore how polypharmacy was assessed in primary care, and (c) seek tested interventions that address polypharmacy^9^. 

Additionally, studies obtained from primary sources, represented by original scientific articles, surveys that have shown data on the occurrence of DDI and ADR in older adults (≥ 60 years of age), female and male sex, were on multiple medications (polypharmacy) were selected. The following were excluded from this review: articles on drug-disease, drug-food, drug-alcohol interactions and drug-nutritional status, abstracts, case study articles, news, commentary, reflection, systematic review articles, clinical updates, expert opinion, studies concomitantly involving child (birth - 18 years), adults (≥19 years), middle aged (≥45 years) and older people (≥ 60 years), research with qualitative approach, editorials, consensus, study protocols, clinical guidelines, reviews, short communication, monographs, theoretical studies and economic evaluation studies and articles published before 2008.

The following bibliographic databases were researched: International Medical Literature Analysis and Retrieval System Online (MEDLINE) via PubMed and EMBASE. In these databases were used Medical Subject Headings Terms (MeSH) and *Emtree* terms. The main descriptors adopted in the search strategy for primary studies were: older adults, polypharmacy, drug interactions, adverse drug reactions and aged, combined using the Boolean operators AND and OR.

After searched, all articles were screened by reading their title, abstract and, when necessary, the content briefly, and, thereby, identifying those papers potentially addressing the topic. The selected articles were analyzed initially and in a second stage, they were read in more detail regarding their content. Finally, the selected articles had their data synthesized. To summarize the data of the selected articles and aiming to ensure that all relevant information was extracted, we applied to each study a validated instrument by Ganong(10). 

In order to determine the relevance of articles captured in the searched databases, two examiners performed the synthesis of the data of interest independently which was followed by the thematic analysis of the papers. Each item synthesized/recorded in the instrument was filed in Microsoft Word(r) 2007, generating a database. All disagreements were resolved by discussion.

The results and data analysis are presented in descriptive form.

## Results

**Figure 1:**
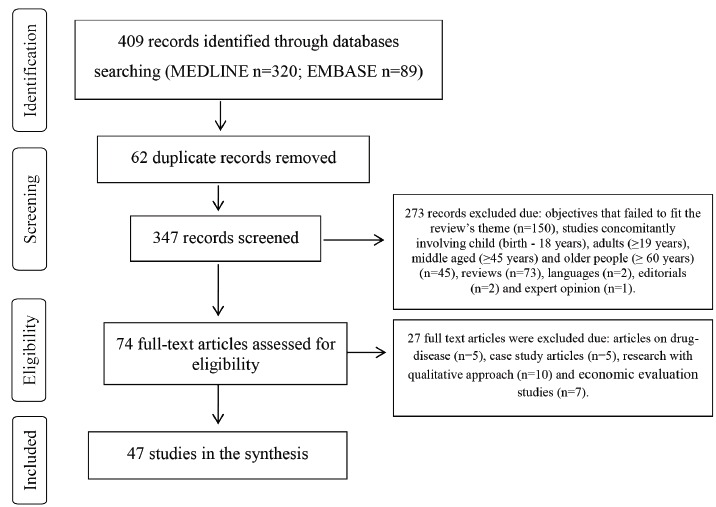
Flow diagram of the articles screened, assessed for eligibility, included and excluded. London, United Kingdom, 2013

A total of 409 references were identified and forty-seven were included in the final analysis. For details, see the flow diagram (Figure 1).

The articles analyzed were from different countries: 9 from (19.1%) the United States, 7 (14.9%) from Canada, 6 (12.8%) from Brazil, 4 (8.5%) from Ireland, 3 (6.4%, respectively) from Belgium and India, 2 (4.3%, respectively) from Australia, Croatia, Spain, Norway and 1 (2.1%, respectively) from France, Indonesia, Netherlands, Singapore, Switzerland, Sweden, Taiwan. Regarding language, 46 (97.9%) articles were written in English and only one (2.1%) in Spanish. Descriptive and analytical studies have been included: cross-sectional (n=9; 19.1%), followed by cohort studies (n=7; 14.9%), nested case-control (n=6; 12.8%) and prospective and prospective observational (n=5; 10.6%, respectively), among others. Considering all the 47 articles examined, a total of 14,624,492 older adults/patients (≥ 60 years) were included, predominantly of hospitals (n=22 articles; 45.8%). Concerning the authorship of publications, 31 (66.0%) are of multidisciplinary researchers, 8 (17.0%) from pharmacists, 7 (14.9%) from physicians and 1 (2.1%) from dentists.

**Figure 2:**
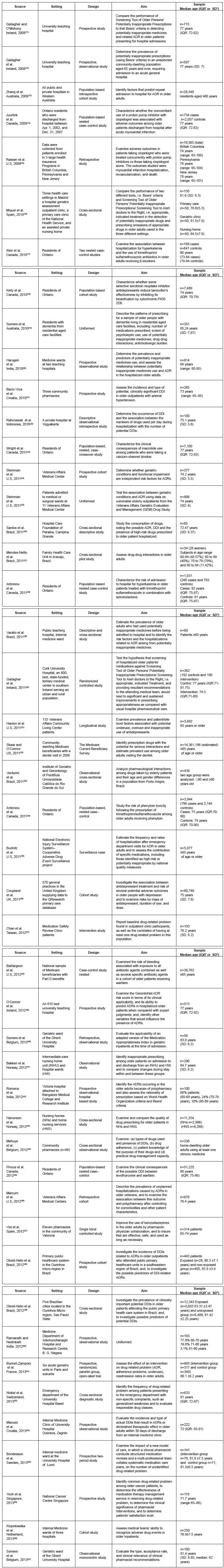
Articles revised according to their characteristics. Brasilia, Federal District, Brazil, 2014

*IQR - Interquatile Range; †SD - Standard Derivation

**Figure 3:**
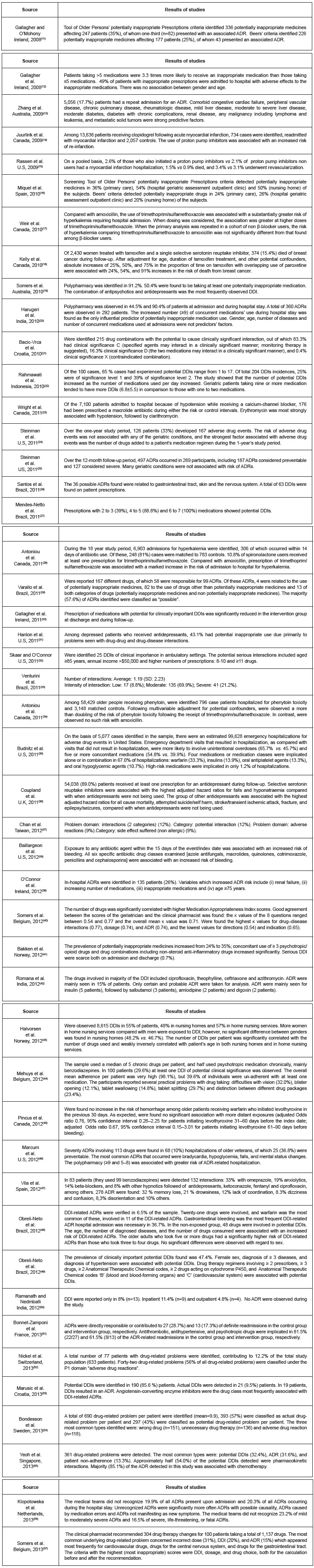
Summary of results of studies on potential DDI and ADR. Brasilia, Federal District, Brazil, 2014

Of the 47 records resulting from the search strategies, 24 (51.1%) full-text articles were retrieved for review concerning ADR^11^ ^-^ ^18^ ^,^ ^20^ ^,^ ^24^ ^-^ ^25^ ^,^ ^28^ ^-^ ^29^ ^,^ ^34^ ^-^ ^36^ ^,^ ^38^ ^-^ ^40^ ^,^ ^46^ ^,^ ^51^ ^-^ ^52^ ^,^ ^54^ ^,^ ^56^ **^)^** , 14 (29.8%) DDI^21^ ^-^ ^23^ ^,^ ^27^ ^,^ ^30^ ^-^ ^33^ ^,^ ^41^ ^,^ ^43^ ^-^ ^45^ ^,^ ^49^ ^,^ ^53^ and nine (19.1%) are related to both DDI and ADR^19^ ^,^ ^26^ ^,^ ^37^ ^,^ ^42^ ^,^ ^47^ ^-^ ^48^ ^,^ ^50^ ^,^ ^55^ ^,^ ^57^, according to Figures 2 and 3.

## Discussion 

To the best of our knowledge, this is the first integrative review examining studies published between 2008-2013 on the occurrence of DDIs and ADRs specifically among older adults. This study helps to demonstrate that the issue in focus is a prevalent problem in various countries. However, generalizing the results of this review is difficult because of the multiplicity of methods and sample sizes of several studies and the diversity of locations they were conducted. Some aspects are highlighted.

Initially, different definitions of polypharmacy were utilized in the studies analyzed such as ≥3^33^, ≥5^19^ ^-^ ^20^ ^,^ ^26^ ^-^ ^27^ ^,^ ^29^ ^,^ ^31^ ^,^ ^37^ ^,^ ^42^ ^,^ ^44^ ^,^ ^46^ ^,^ ^51^, ≥6^52^ or ≥10^11^ ^-^ ^12^ ^,^ ^15^. In thirty-one studies there was no definition of polypharmacy^13^ ^-^ ^14^ ^,^ ^16^ ^-^ ^18^ ^,^ ^21^ ^-^ ^25^ ^,^ ^28^ ^,^ ^30^ ^,^ ^32^ ^,^ ^34^ ^-^ ^36^ ^,^ ^38^ ^-^ ^41^ ^,^ ^43^ ^,^ ^45^ ^,^ ^47^ ^-^ ^50^ ^,^ ^53^ ^-^ ^57^. Therefore, there is a need for a clear cut-off point that defines polypharmacy worldwide. A definition focusing on whether the medication is clinically indicated may be more appropriate^9^ ^)^ than the number of ingested medicines.

DDI and ADR are frequently the end result of polypharmacy as shown in the studies analyzed^11^ ^-^ ^57^, and are associated with others predictors, for example: sex differences^24^ ^,^ ^33^; sex of patients, alcohol consumption and smoking habits^29^; increased age^39^ ^,^ ^49^; diagnoses of diseases and multiple comorbidities^21^ ^,^ ^25^ ^,^ ^48^ ^-^ ^49^ ^,^ ^53^; use the specific types drugs, such as patients using clopidogrel with proton pump inhibitors^14^ ^-^ ^15^, tamoxifen^18^, co-prescription of macrolide antibiotics and calcium-channel blockers^23^,trimethoprim/sulfamethoxazole^17^ ^,^ ^28^ ^,^ ^34^,antidepressants^31^ ^,^ ^36^,warfarin^38^ ^,^ ^45^, benzodiazepines^47^; also, cognitive impairment and various functional problems that affect practical drug management capacity^24^ ^-^ ^25^; living situation^19^; access to health care, prescription of drug therapy regimens by two or more prescribers^49^; and educational status^31^ ^,^ ^44^ ^,^ ^50^ . 

Non-adherence to treatment is a common problem in older adults. DDIs and ADRs during hospitalization have been reported to be associated with non-adherence, which are also common among older adults who are discharged from hospital and are using several drugs for their chronic diseases. Studies examining readmissions due to DDIs and consequent ADRs were also performed^44^ ^,^ ^51^ ^,^ ^55^. Therefore, early detection and recognition of clinically important interactions by healthcare professionals are vital for monitoring the occurrence of DDIs and ADRs in the continuum of health care.

Older adults usually do better use of medicines when their care is managed by a multidisciplinary team, consisting of a physician (geriatrician), clinical pharmacist and nurse. The involvement of a dentist in this team seems to be relevant, as demonstrated in a study^32^.

Inappropriate prescription, with misuse of medication, poor quality of doctors' choices of prescriptions, over-prescription of drugs, additional medicines prescribed to treat side effects, and poor team-patient relationships may increase the chances of occurrences of DDI and ADR. By combining their knowledge and skills, a comprehensive plan and dosage adjustments can be developed to enable best pharmacotherapy while the risks of DDI and ADR are reduced. An efficient communication between these professionals and coordination across multiple prescribers is crucial for success. Moreover, educational programs should be conducted to improve the habit of prescribing rationally. Equally, patient education at discharge and follow-up is also very important.

Use of multiple medications increases the risk of inappropriate prescribing. Different interventions to optimize prescribing appropriateness in older adults, for example, the Beers' criteria, most often used in the United States^12^ ^,^ ^16^ ^,^ ^20^ ^,^ ^47^, the validated Screening Tool of Older Persons' Potentially Inappropriate Prescriptions (STOPP) and Screening Tool to Alert doctors to the Right, i.e. appropriate, indicated Treatment (START) criteria^11^ ^,^ ^16^ ^,^ ^30^ in the Ireland and United Kingdom, the Norwegian General Practice criteria (NORGEP)^41^ ^,^ ^43^, and the instrument Medication Appropriateness Index (MAI)^40^ ^,^ ^57^ have been explored in studies. The studies reviewed indicated that STOPP/START criteria identified a higher proportion of inappropriate prescription than Beers' criteria. 

This review showed that there are different frequencies and types of DDIs and ADR, which are drug-related problems, associated with different classes of drugs. In daily care practice, the correct diagnosis of these problems requires skill and expertise of the multidisciplinary team, especially when older adults present themselves with nonspecific complaints and manifestations. To recognize and diagnose this undesirable outcome, goals should be set in the health care service, highlighting the role of the clinical pharmacist, who uses interventions for identification and minimization the drug-related problem (DRP), as demonstrated in studies^4^ ^-^ ^57^. 

Balancing the risks and benefits of multiple drug therapies may be useful in the establishment of rational interventions for the safe use of drugs. Accordingly, the use of technologies in the monitoring of DDIs and recognition of ADRs, such as computer-based screening, could help practitioners to recognize potential and clinically significant interactions and adverse events. The software must have high sensitivity and specificity, and high positive and negative predictive value. In the same way, the advantage of utilizing computerized databases for reviewing the medications' prescriptions is evident.

Thus, the reviewed studies reinforced the notion that polypharmacy is multifactorial and is associated with negative health outcomes, as reported previously in two studies reviewed^8^ ^-^ ^9^, and in the article of experts opinion that present information specifically of 12 studies about DDI and ADR^58^.

One aspect noted and that needs to be investigated further, relates to examine how self-medication with over-the-counter drugs and complementary medications contribute to increases the risk of DDIs and ADRs, hospitalization and death of older adults. Other gap noted in literature relates to the methods utilized by primary care providers when assessing polypharmacy. Additionally, little information is available about the incidence/prevalence of DDIs and ADRs among older adults in developing countries.

So, this review points out the relevance of conducting more studies to explore different aspects, considering the need to develop preventive practices to guarantee the safety of older adults with regard to DDIs and ADRs. 

### Limitations and strength of study

Firstly, publication bias which cannot be measured due to both language and database limitations. Selection bias may have occurred because unpublished research and/or development were not selected. Possibly, another limiting aspect is the lack of methodological assessment and risk of bias due to the heterogeneity of the reviewed studies. A major strength of this study is rigorous review of the literature, specifically on the consequences of DDIs and ADRs related to polypharmacy, with diverse methodological designs, including studies published worldwide.

### Conclusion and implications for advanced practice in geriatric nursing 

This study has identified that early detection and recognition of clinically important DDI and ADR by healthcare professionals are vital to identify patients who are at higher risk for such events and require more cautious pharmacotherapy management to avoid negative outcomes. Thus, the potential risk to DDI and ADR can be managed by professionals with appropriate prescriptions, monitoring, and patient education in the continuum of care of older adults, i.e. through best practices.

In this sense, the professionalization on advanced nursing practice is essential, as a requirement for acquisition of knowledge, skills training and skills for making safe and effective care decisions, for example, aimed at health care of older adults commonly exposed to polypharmacy. Thus, this integrative review can help to increase awareness and discussion, to implement universal health coverage and universal access to health care of the older adults in order to guarantee the quality of care by geriatric nurses.
